# Intragastric and Intranasal Administration of *Lactobacillus paracasei* NCC2461 Modulates Allergic Airway Inflammation in Mice

**DOI:** 10.1155/2012/686739

**Published:** 2012-03-21

**Authors:** Céline Pellaton, Sophie Nutten, Anne-Christine Thierry, Caroline Boudousquié, Nathalie Barbier, Carine Blanchard, Blaise Corthésy, Annick Mercenier, François Spertini

**Affiliations:** ^1^R&D Laboratory of the Division of Immunology and Allergy, Centre Hospitalier Universitaire Vaudois, 1011 Lausanne, Switzerland; ^2^Nutrition and Health Department, Nestlé Research Centre, 1000 Lausanne, Switzerland

## Abstract

*Introduction*. Preclinical and clinical evidences for a role of oral probiotics in the management of allergic diseases are emerging. *Aim*. We aimed at testing the immunomodulatory effects of intranasal *versus* intragastric administration of *Lactobacillus paracasei* NCC2461 in a mouse model of allergic airway inflammation and the specificity of different probiotics by comparing *L. paracasei* NCC2461 to *Lactobacillus plantarum* NCC1107. *Methods*. *L. paracasei* NCC2461 or *L. plantarum* NCC1107 strains were administered either intragastrically (NCC2461) or intranasally (NCC2461 or NCC1107) to OVA-sensitized mice challenged with OVA aerosols. Inflammatory cell recruitment into BALF, eotaxin and IL-5 production in the lungs were measured. *Results*. Intranasal *L. paracasei* NCC2461 efficiently protected sensitized mice upon exposure to OVA aerosols in a dose-dependent manner as compared to control mice. Inflammatory cell number, eotaxin and IL-5 were significantly reduced in BALF. Intranasal supplementation of *L. paracasei* NCC2461 was more potent than intragastric application in limiting the allergic response and possibly linked to an increase in T regulatory cells in the lungs. Finally, intranasal *L. plantarum* NCC1107 reduced total and eosinophilic lung inflammation, but increased neutrophilia and macrophages infiltration. *Conclusion*. A concerted selection of intervention schedule, doses, and administration routes (intranasal versus intragastric) may markedly contribute to modulate airway inflammation in a probiotic strain-specific manner.

## 1. Introduction

Respiratory allergy results from inappropriate adaptive immune responses to ubiquitous, otherwise most often innocuous environmental proteins. The immunological hallmarks of respiratory allergy are mainly characterized by the aberrant production of Th2 cytokines (IL-4, IL-5, IL-13) and the induction of IgE and chemokines involved in the recruitment of lymphocytes and eosinophils into the lungs. While proteins targeted by airway allergic responses represent a tiny fraction of airborne proteins humans are exposed to, allergenicity is a quite common phenomenon. Genetic predisposition has a key role in allergy expression, but substantial evidences have emerged emphasizing that the microbial environment is a major player in maintaining a Th1/Th2 homeostasis. This concept relies on the “hygiene hypothesis” stating that avoidance of microbial exposure during early infancy increases the risk of developing allergic diseases [1–3].

Probiotics are defined as live microorganisms which confer a health benefit on the host when administered in adequate amounts [4]. Originally thought to be due to improvements in the intestinal microbial balance, probiotics beneficial effects may also stem from their capacity to modulate the host immune response [5, 6]. Recent clinical trials, epidemiological studies, and animal experiments have suggested that probiotics may contribute to suppress the development of allergic responses. In humans, most successes have been obtained in primary prevention of atopic eczema, but a limited number of studies also provided evidence for a beneficial effect of different probiotics in the management of other allergic diseases such as allergic rhinitis [5]. A few studies conducted in children and adults with allergic rhinitis suggested a beneficial effect of specific probiotic strains (*Lactobacillus casei*, *Bifidobacterium longum*, or *Lactobacillus paracasei*) [7–9]. Several reports have also shown that probiotic supplementation was effective at preventing asthma symptoms when administered very early in life [10–12]. To our knowledge, only few studies showed that oral treatment with a *Lactobacillus reuteri* strain inhibited the allergic airway response in adult mice [13]. These studies derived from the observation that endotoxin and/or bacterial exposure early in life, or during pregnancy, protects the offspring against allergen-induced airway disease [14, 15]. Probiotics may modulate allergy through immune deviation toward a Th1 immune response or induction of regulatory T cells [16]. These modes of action may overlap or differ depending on the models used, the intrinsic properties of the probiotics utilized and their TLR ligand expression. 

*Lactobacillus paracasei* NCC2461 is a probiotic strain that was selected on the basis of its safety, its industrial properties (yield, stability), and its *in vitro* immune modulation profile. This strain has been shown to produce antimicrobial metabolites and to increase Th1 cell-dependent immune system activation as well as regulatory T cells development [5, 6, 17–19].

We demonstrate in this study that the schedule (prior to, during, or after OVA sensitization or during OVA aerosol challenges), the administration routes (intranasal versus intragastric) and the characteristics of the strain administered (*L. paracasei* NCC2461 versus *L. plantarum* rather than *Lactobacillus* NCC1107, used here as a negative control according to previous *in vitro* screening and *in vivo *testing [20] were key parameters of the modulation of murine airway inflammation by probiotics.

## 2. Material and Methods

### 2.1. Experimental Airway Inflammation

Four-week-old female BALB/c mice (H-2^d^) were obtained from Harlan (AD Horst, The Netherlands) and used at the age of 6–8 weeks. They were maintained under standard housing conditions on ovalbumin- (OVA-) free diet and water *ad libitum*. Mice were sensitized twice at day 0 and 14 by intraperitoneal (i.p.) injections of 10 *μ*g of OVA (Fluka, Buchs, Switzerland) adsorbed on 1 mg Alum (Sigma Chemicals, St-Louis, MO, USA) [21]. On days 26, 28, and 30, animals were exposed to a single aerosol of OVA in PBS (0.25%). Aerosolization generated by a nebulizer (De Vilbiss, Sunrise medical GmBH, Germany) was performed with a maximum of 12 mice placed in a 30 × 30 × 12 cm plexiglas chamber and exposed for 20 min. Animals were sacrificed on day 33.

### 2.2. Probiotics Administration 

**Figure 1:**
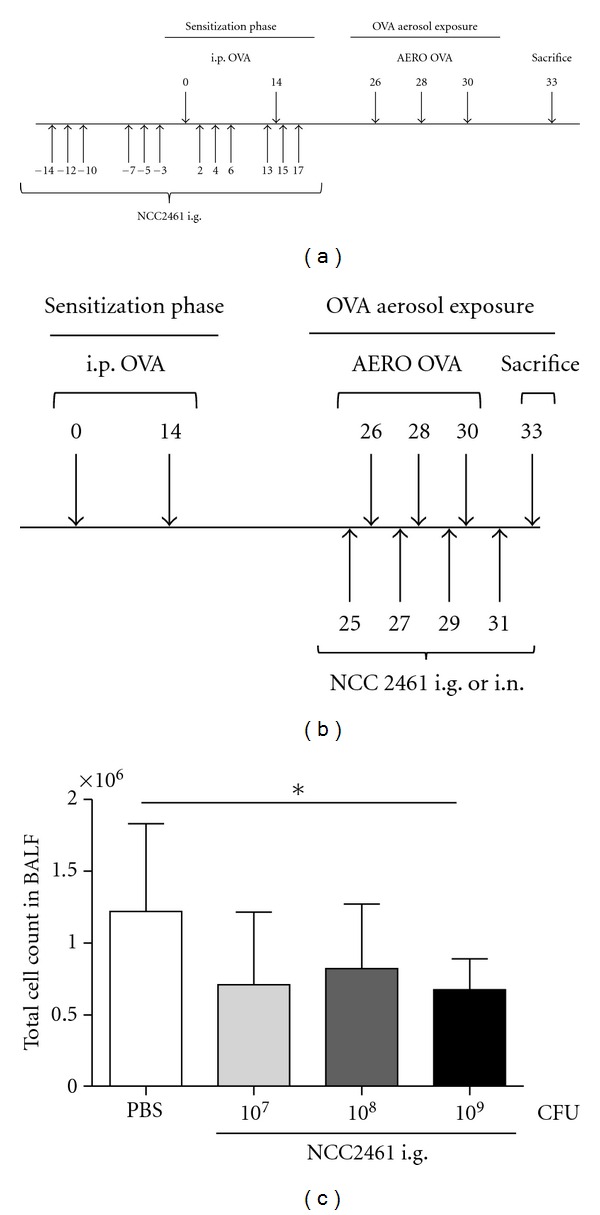
Protocols of probiotic strains administration and subsequent effect of intragastric *L. paracasei *NCC2461 on total cell number in BALF of OVA-challenged mice. Mice were sensitized intraperitoneally (i.p.) twice with OVA and subsequently challenged with OVA aerosol 3 times. Mice received NCC2461 intragastrically (i.g.) 12 times before, after, and in between the 2 i.p. sensitizations (a) or 4 times before, after and in between each OVA challenge (b). Total cell count in the BALF (*n* = 10) mice per group. In this representative experiment, data are expressed as mean ± SD; *P < 0.05 (c).

The strains *L. paracasei* NCC2461 (CNCM I-2116; ST11; Nestlé, Switzerland) and *L. plantarum* NCC1107 are part of the Nestlé Collection and were provided by Nestlé. Probiotic bacteria at a dose of 10^9^ CFU (unless other dose specified) were applied intragastrically (i.g.) or intranasally (i.n.), either 12 times during sensitization phase (Figure 1(a)) or 4 times every other day, i.e. the days without OVA aerosols (days 25, 27, 29, 31) (Figure 1(b)). Intragastric administration of probiotics in PBS was done using a stainless steel feeding tube in a volume of 100 *μ*L. Intranasal administration of 20 *μ*L probiotics in PBS or PBS only was performed under light anesthesia with halothane (Halocarbon B. P., Arovet A. G., Zollikon, Switzerland). Material was spontaneously inhaled by the animal. 

### 2.3. Histology

Whole lungs were fixed by inflation and submersion in 10% buffered formalin, embedded in paraffin, and then sectioned. Histopathologic study was made using Hematoxylin and Eosin (H&E)—and Periodic Acid Schiff (PAS)—stained lung sections. Representative pictures of H&E staining were taken. The percentage of PAS staining positive cells in small- and medium-size airways were counted out of all the available epithelial cells present on each section.

### 2.4. BALF Collection and Cytospin Preparation

At the time of sacrifice, animals were anesthetized intraperitoneally (i.p.) with 3 mg thiopental sodium (Trapanal, Altana pharma GmbH, Konstanz, Germany), trachea was cannulated, and bronchoalveolar lavage (BAL) was performed by injecting 3 mL PBS (6 × 500 *μ*L) into the lungs. BAL fluid (BALF) was immediately stored on ice. Approximately, 10^5^ cells in 100 *μ*L were centrifuged on glass plates (Cytospin, Shandon scientific, Cheshire, UK) and then stained with DiffQuik according to manufacturer's recommendations (Baxter Dade, Dudingen, Switzerland). A differential count of 200 cells was performed using standard morphological criteria.

### 2.5. IgE Determination by ELISA in Sera

Serum IgE levels were determined by ELISA as described [22, 23]. Briefly, after blocking with 1% BSA, 1 : 20 dilutions of mouse serum (50 *μ*L) were incubated in 96-well Nunc Maxisorp immunoplates (Life Technologies, Basel, Switzerland) previously coated with 5 *μ*g/mL OVA. After three washes in PBS-Tween 0.02%, plates were incubated with 2 *μ*g/mL biotinylated rat anti-mouse IgE mAb (PharMingen, BD-Biosciences, San Diego, CA, USA) for two hours and revealed with alkaline phosphatase. Purified mouse IgE (PharMingen) was used as a standard. Results were expressed in ng/mL. 

### 2.6. Quantification of Cytokines and Chemokine by ELISA

Lungs were harvested, snap-frozen in dry ice, and kept at −80°C until use. Frozen lungs were homogenized in a Dounce tissue grinder with 1 mL PBS containing protease inhibitors (Complete, Roche Diagnostics GmbH, Mannheim, Germany) and centrifuged for 4 min at 1640 × g. Cytokines and chemokines were measured in supernatants (50 *μ*L) by ELISA according to the manufacturer (PharMingen/BD Biosciences, San Diego, USA, for IL-5 or R&D Systems, Minneapolis, USA, for eotaxin-1). 

### 2.7. Lungs Cells Isolation and T Regulatory Cells Staining

Lungs were minced in NaCl 0.9% and incubated 20 minutes at 37°C in the presence of 0.2 *μ*g/mL Liberase (Roche Diagnostic GmbH, Mannhein, Germany), 0.1 *μ*g/mL DNase (Sigma Chemicals, St-Louis, MO, USA), and 5 mM CaCl_2_ (Sigma Chemicals, St-Louis, MO, USA) at 37°C. They were then homogenized on a 40 *μ*m cell strainer (BD Falcon, Basel, USA), rinsed with Dulbecco's modified Eagle's medium supplemented with 10% FCS and centrifuged. Red blood cells were lysed in lysis buffer (0.15 M NH_4_CL, 0.01 M KHCO_3_), washed, and resuspended in FACS buffer (PBS, 1% BSA, 0.01% NaN_3_). Cells were finally incubated with the following antibodies: anti-CD4 PerCP (1/200, BDPharmingen), anti-CD25PE (1/300, BD Pharmingen), staining kit FJK-16s (eBioscience, San Diego, Ca, USA), for 20 min on ice. Foxp3 intracellular staining was performed using anti-mouse/rat Foxp3 staining set (FJK-16s, eBioscience, San Diego, CA, USA) according to manufacturer's protocol. Flow cytometry acquisitions were performed on a FACScalibur (BD Biosciences) and analyzed using FlowJo software (Tree Star Inc., Ashland, OR, USA).

## 3. Results 

### 3.1. *L. paracasei* NCC2461 Reduces Inflammatory Cell Recruitment into BALF when Administered by the Intragastric Route during Allergen Challenges

To investigate the *in vivo* immunomodulatory properties of *L. paracasei* NCC2461, several protocols were used to evaluate the protective effect of this strain NCC2461 during the different phases of the OVA allergic airway inflammation model. Bacteria were administered to mice during the sensitization phase (Figure 1(a)) or at the time of aerosol exposure (Figure 1(b)). The intragastric supplementation of *L. paracasei* NCC2461 during the aerosol exposures significantly reduced the total cell number in the BALF (Figure 1(c)). An intragastric dose of 10^7^ CFU *L. paracasei* NCC2461 tended to downregulate inflammatory cell recruitment into the BALF whereas a higher i.g. dose of 1 × 10^9^ CFU *L. paracasei* NCC2461 significantly reduced cell recruitment as compared to PBS control, from 1.22 × 10^6^ ± 6.11 × 10^5^ (mean ± SD) to 6.75 × 10^5^ ± 2.15 × 10^5^ total cell number, that is a 45% decrease (*P* < 0.05) (Figure 1(c)). This protective effect was not significant when *L. paracasei* NCC2461 was administered i.g. during the sensitization phase with OVA (data not shown), suggesting a better protective action of this strain when administered in already sensitized animals, during the OVA aerosol challenges. Specific IgE levels in plasma were not affected by the administration of *L. paracasei* NCC2461 in the two experimental settings (data not shown). Globally, these results indicated that *L. paracasei* NCC2461, administered intragastrically at a dose of 1 × 10^9^ CFU, significantly impaired inflammatory cell recruitment into BALF when administrated to mice during the OVA aerosol exposure phase. 

### 3.2. Intranasal *L. paracasei* NCC2461 Administration is More Efficient Than Intragastric Administration in Reducing IL-5 and Eotaxin Production in Lungs

**Figure 2:**
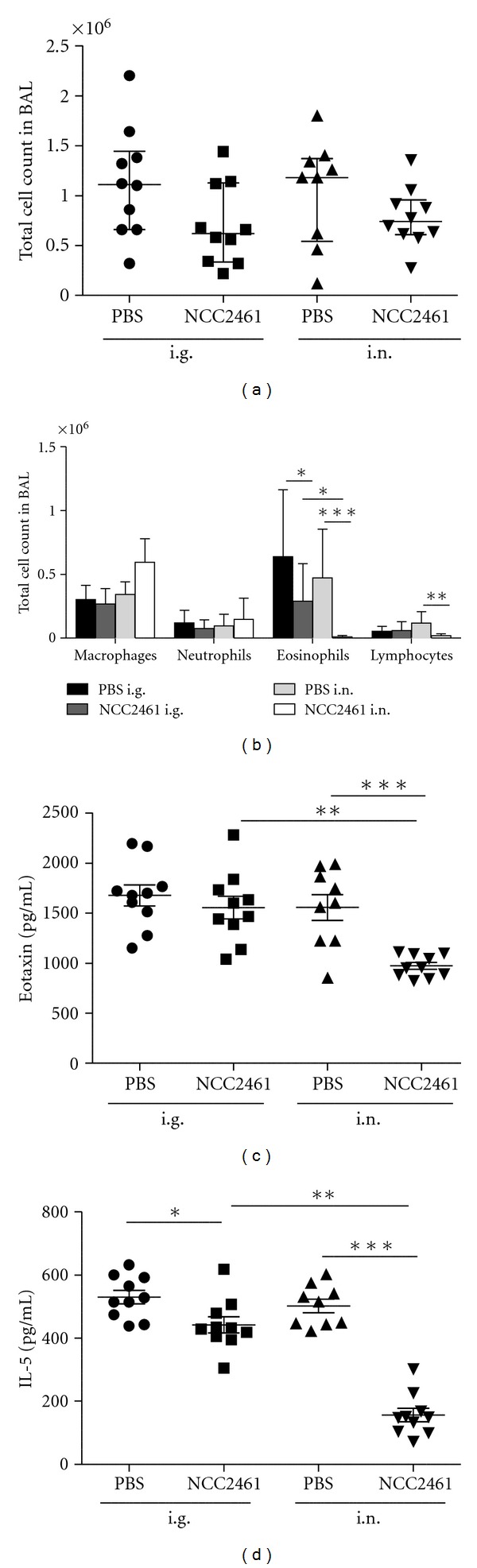
Effect of intragastric versus intranasal *L. paracasei* NCC2461 administration on lung airway inflammation. *L. paracasei* NCC2461 (1 × 10^9^ CFU) or PBS were either administered intragastrically (i.g.) or intranasally (i.n.) in OVA-challenged mice. Total cell counts (a) and differential cell counts (b) in the BALF. Eotaxin (c) and IL-5 levels (d) in lung homogenate were quantified by ELISA. Histograms are mean ± SD obtained from one representative experiment, *n* = 10 mice per group; *P < 0.05, **P < 0.005, ***P < 0.0005.

 We next tested whether the administration of *L. paracasei* NCC2461 (at a dose of 1 × 10^9^ CFU) via the nasal *versus* gastric route during OVA aerosol exposure would be able to reduce cell recruitment into BALF (Figure 1(b)). In agreement with the results obtained after intragastric administration of *L. paracasei* NCC2461 (Figures 1(c) and 2(a)), total cell count in BALF was decreased in mice given *L. paracasei* NCC2461 intranasally (i.n.) (Figure 2(a)). Differential cell count in BALF was analyzed and a significant 2.1-fold drop in eosinophil numbers was observed in mice treated by intragastric *L. paracasei* NCC2461 gavage (6.42 × 10^5^ ± 5.23 × 10^5^ versus 2.93 × 10^5^ ± 2.92 × 10^5^) (Figure 2(b)). Macrophages, neutrophils and lymphocytes numbers were not significantly altered under these conditions. Interestingly, intranasal administration of *L. paracasei* NCC2461 was able to dramatically decrease eosinophilic recruitment into the BALF by 37.8-fold as compared to PBS-treated group (1.26 × 10^4^ ± 1.16 × 10^4^ versus 4.76 × 10^5^ ± 3.81 × 10^5^, *P* < 0.0005). Lymphocytes were also significantly decreased (5.4-fold, *P* < 0.005) in *L. paracasei* NCC2461 intranasally treated mice compared to PBS-treated mice. OVA-sensitized mice given *L. paracasei* NCC2461 intranasally showed a significant drop in IL-5 and eotaxin (Figures 2(c) and 2(d)) production in lung homogenates. During such a short observation period, no effect of i.n. administration of the strain was observed on specific IgE quantified in serum (data not shown). Altogether these results suggested that the intranasal route was more efficient than the intragastric administration in downregulating key inflammatory mediators for eosinophil recruitment and survival into lung airways, that is eotaxin and IL-5, although final total cell recruitment into BALF was similar. 

### 3.3. *L. paracasei* NCC2461 Is More Efficient Than *L. plantarum* NCC1107 to Reduce Airways Inflammation

**Figure 3:**
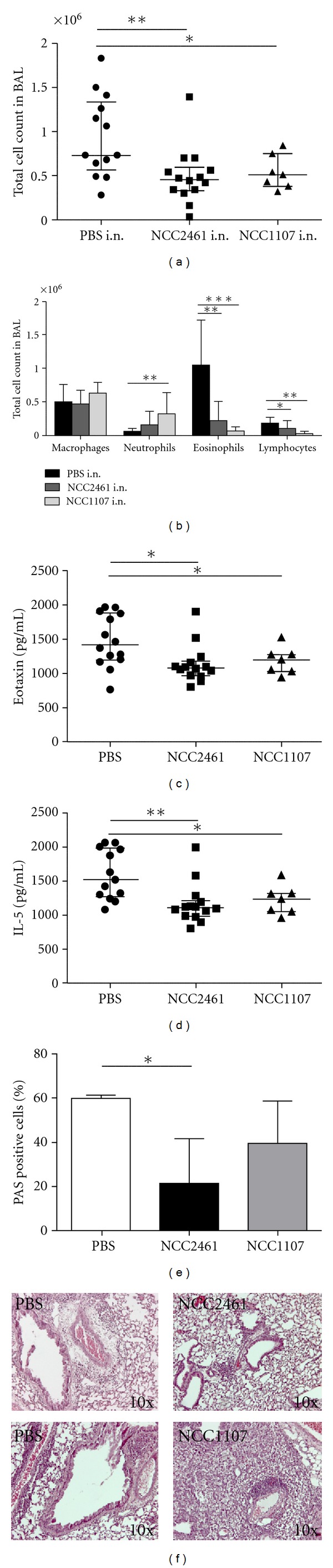
Effect of intranasal *L. paracasei* NCC2461 and *L. plantarum* NCC1107 on lung airway inflammation. *L. paracasei* NCC2461 (1 × 10^9^ CFU), *L. plantarum* NCC1107 (1 × 10^9^ CFU), or PBS were administered intranasally (i.n.) in OVA-challenged mice. Total (a) and differential (b) cell counts were performed in the BALF. Eotaxin (c) and IL-5 levels (d) in lung homogenate were quantified by ELISA. Lung sections were PAS stained (results are expressed as % of PAS positive cells (e)) and H&E stained (f), reflecting the severity and localization of inflammation (magnification ×10). In this representative experiment, histograms are mean ± SD from 10 animals; *P < 0.05, **P < 0.005, ***P < 0.0005.

We next aimed at testing the specificity of *L. paracasei* NCC2461 in reducing airways inflammation by comparing *L. paracasei* NCC2461 to *L. plantarum* NCC1107 administered at the same dose of 1 × 10^9^ CFU. To this end we selected the intranasal route of application as it induced more prominent immune modulation. As previously observed with *L. paracasei* NCC2461, total cell recruitment in the BALF was also significantly reduced when mice were treated with *L. plantarum* NCC1107 during OVA aerosol exposure (*P* < 0.05) as compared to PBS (9.81 × 10^5^ ± 6.36 × 10^5^ with NCC2461 treatment, 1.08 × 10^6^ ± 3.84 × 10^5^ with NCC1107 treatment and 1.88 × 10^6^ ± 9.27 × 10^5^ with the PBS treatment, Figure 3(a)). Both *Lactobacillus *strains induced a decrease in eosinophils and lymphocytes recruitment into the lungs (Figure 3(b)) that was accompanied and supported by a reduced production of IL-5 (Figure 3(c)) and eotaxin (Figure 3(d)). However, only *L. plantarum* NCC1107 induced a significant neutrophil influx (4.8-fold, *P* < 0.05) into BALF, not observed after *L. paracasei* NCC2461 administration (Figure 3(b)). These differences were also illustrated histologically. A significant reduction of PAS-staining positive cells was observed in the lungs of NCC2461-treated mice while no significant changes were observed in NCC1107-treated mice (*P* = 0.114; Figure 3(e)). Based on H&E staining, the lungs of NCC2461-treated animals were presenting less perivascular inflammation than the PBS control group (Figure 3(f)). It is interesting to note that, while the total BAL cell count and absolute BAL eosinophilia were reduced in the NCC1107-treated mice BAL, a profound tissue lung inflammation, not only restricted to the perivascular zone, was observed in this group (Figure 3(f)). Similarly to what was observed after *L. paracasei* NCC2461 i.n. administration, levels of specific IgE in plasma were similar in the group of mice administered i.n. with *L. plantarum* NCC1107 and in the control group (data not shown). Taken together these results suggested that *L. paracasei* NCC2461 and *L. plantarum* NCC1107 have different modulatory competences on allergic lung airway inflammation, and that *L. plantarum* NCC1107 may, in respect to neutrophil recruitment, even lead to enhanced airways inflammation.

### 3.4. *L. paracasei* NCC2461 Increases Regulatory T Cells in the Lungs

**Figure 4:**
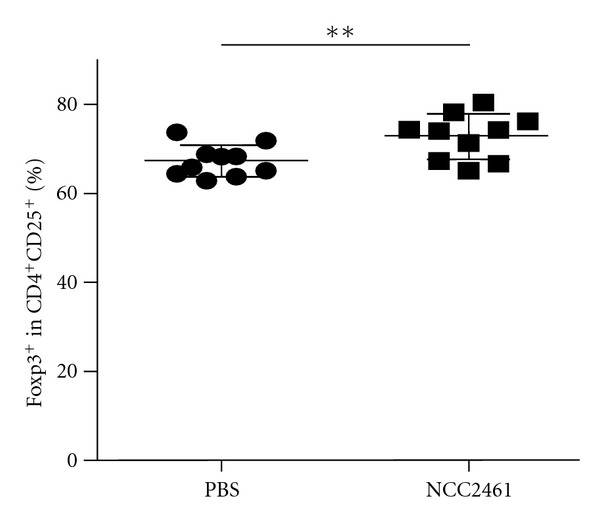
Percentage of FoxP3 positive cells in the CD4^+^CD25^+^ T cell population in the lung of mice treated intranasally with PBS or with NCC2461. One representative experiment out of two independent experiments. **P < 0.005.

To better understand the beneficial role of intranasal administration of *L. paracasei* NCC2461 in this respiratory allergy mouse model, we investigated the presence of regulatory T cells in lungs of mice treated with NCC2461. Although the difference was small, the percentage of CD4^+^CD25^+^ cells expressing Foxp3 was significantly higher in NCC2461 treated mice than in PBS controls (Figure 4). This decrease may be associated with the observed downregulation of inflammatory markers and cell infiltrates in the lungs and BALF of probiotic-treated mice.

## 4. Discussion

Probiotic supplementation for prevention or reduction of allergic symptoms is well documented, but conflicting results have been reported so far. Here, we demonstrate that the probiotics administration schedule, dose, strain, and routes were key parameters for a reliable downregulation of airways allergic inflammation. The decrease in pulmonary cell infiltrates observed in our model was marked and consistent across experiments, and the downregulation of inflammatory markers (IL-5, eotaxin) quite substantial. These results can be compared to the effect obtained with steroids in human asthma [24]. In humans, however, trials involving probiotics have often led to mild and variable effects on allergic inflammation, which may reflect a greater impact of confounding factors as compared to a “controlled” mouse model. It is however important to keep in mind that probiotics in humans were primarily investigated to delay onset and to decrease incidence and prevalence of allergy symptoms, or ultimately to alleviate them, and to improve quality of life of patients. In this study, probiotics were able, in a therapeutic manner, to significantly modulate allergic inflammatory markers such as lung eosinophilia and lymphocyte recruitment as well as lung eotaxin and IL-5 production. Of note, different routes of probiotic administration generated different immune effects; indeed the application of probiotics directly to the respiratory mucosa was more effective at decreasing key allergic features (IL-5, eotaxin) than the intragastric route. This could result from the fact that the nasal cavity corresponds to a less complex ecosystem than the gastro-intestinal tract. Moreover, the capacity to modulate inflammation appeared to differ between the two strains studied. 

Several studies have shown that probiotic supplementation was effective in preventing allergic airway symptoms when administered early in life [11, 12, 17, 18]. Bacterial exposure early in life, or during pregnancy, has been shown to protect the offspring against allergen-induced airway disease [14]. Another study described the therapeutic potential of oral treatment with a strain of *Lactobacillus reuteri* on allergic airway response in mice [13]. Interestingly, the oral administration of another strain (*Lactobacillus salivarius*) in the same animal model did not show any beneficial effect, underlining the specificity of the strain for a given health benefit. In our study as well, the two tested *Lactobacillus* strains had specific properties. While *L. plantarum *NCC1107 was efficient at reducing eosinophil influx into the BALF, it also increased significantly neutrophilia, a phenomenon never observed with *L. paracasei* NCC2461. Interestingly, *L. plantarum *NCC1107 had no effect in another allergy model (intestinal food allergy model) [20].

Probiotics may modulate allergy through immune deviation toward a Th1 immune response or through the induction of regulatory T cells [25]. *L. paracasei* NCC2461 has been widely studied and has been precisely shown to induce the production of immunomodulatory as well as Th1 cytokines *in vivo* and *in vitro* [18]. Here, we demonstrated that *L. paracasei *NCC2461 was less effective at preventing the induction of specific markers of airways allergic inflammation (IL-5, eotaxin) when delivered intragastrically, as opposed to intranasally, during the challenge phase. Nonetheless, the final total inflammatory cell numbers in BALF were similar, suggesting potentially two modulation pathways differing when generated in the respiratory or in the gastrointestinal tract. We also demonstrated that the eosinophilic lung infiltrate was more efficiently reduced when probiotics were administered intranasally as compared to intragastrically. This suggested a direct crosstalk between the nasal and the lung mucosae to efficiently counteract the Th2 response as previously demonstrated [21]. Eotaxin was not significantly decreased after intragastric *L. paracasei *NCC2461 but eosinophilia was nonetheless reduced. This may result from a potential inhibitory action of probiotics on lung eosinophilic recruitment via a systemic effect or by decreasing other chemokines. Finally, in agreement with other studies in spleen [26], or in peribronchial lymph nodes [11], we showed that the CD4^+^CD25^+^Foxp3^+^ T cell population was increased in the lungs of *L. paracasei *NCC2461-treated mice as compared to the lungs of control animals. As also demonstrated in a model of tolerance to OVA via the nasal mucosa, this enhancement of the T regulatory cell subpopulation may contribute to the mechanisms responsible for the anti-inflammatory effect of *L. paracasei *NCC2461 [27]. 

Altogether, these results confirm the potential interest of probiotics in allergy management and the previously known concept that the health benefits delivered by probiotics are highly strain specific. Additionally, their efficacy at a defined site and in a precise model cannot be generalized to all sites, strains or models. As such, while murine models can help selecting probiotics with anti-inflammatory or anti-allergy potential, clinical trials in allergic patients will need to be performed to definitively establish their efficacy and to better understand their specific effects in the human host. Importantly, the potential benefit of *L. paracasei* NCC2461 was supported in humans by two recent pilot clinical trials in which *L. paracasei* NCC2461 consumption was able to decrease inflammatory cell infiltrates in the nasal mucosa of adults suffering from grass pollen-induced allergic rhinitis [8].

## Abbreviations

BALF: Bronchoalveolar lavage fluid
i.n.: Intranasal
i.g.: Intragastric
i.p.: Intraperitoneal.
